# Altered Sex Ratio at Birth with Maternal Exposure to Dioxins in Vietnamese Infants

**DOI:** 10.3390/toxics12040276

**Published:** 2024-04-09

**Authors:** Noriko Kaneko, Muneko Nishijo, Hoa Thi Vu, Tai Pham-The, Thao Ngoc Pham, Nghi Ngoc Tran, Tomoya Takiguchi, Yoshikazu Nishino

**Affiliations:** 1 Department of Nursing, Ishikawa Prefectural Nursing University, Kahoku 929-1210, Japan; phnkaneko2@gmail.com; 2 Department of Public Health, Kanazawa Medical University, Uchinada 920-0293, Japan; ttakiguc@kanazawa-med.ac.jp (T.T.); ynishino@kanazawa-med.ac.jp (Y.N.); 3 Institute of Biomedicine and Pharmacy, Vietnam Military Medical University, Hanoi 12108, Vietnam; vuhoa5593hvqy@gmail.com (H.T.V.); taithuy@kanazawa-med.ac.jp (T.P.-T.); 4 103 Military Hospital, Vietnam Military Medical University, Hanoi 12108, Vietnam; phamngocthaovmmu@gmail.com; 5 Ministry of Health, Vietnam Government, Hanoi 10060, Vietnam; nghi_tranngoc@yahoo.com

**Keywords:** dioxin, sex ratio, breast milk, herbicide, Vietnam

## Abstract

Excess female births (lower sex ratio at birth) associated with paternal exposure to 2,3,7,8-tetrachlordibenzo-p-dioxin (TCDD) have been reported in Italy. However, no significant effects of maternal TCDD exposure on the sex ratio were reported. We investigated the effects of maternal TCDD exposure and the toxic equivalent quantity of polychlorinated dibenzo-p-dioxins/dibenzofurans (TEQ-PCDD/Fs) on the sex ratio at birth in 576 Vietnamese infants from three birth cohorts. TCDD and TEQ-PCDD/Fs in breast milk were stratified (low, mild, moderate, and high) as maternal exposure markers. Logistic regression analysis was used to investigate associations between female birth and dioxin exposure groups after adjusting for confounders. In sprayed and unsprayed areas, adjusted odds ratios (ORs) of female birth (reference: low-TCDD group) were 2.11 in the moderate-TCDD group and 2.77 in the high-TCDD group, which were significantly associated with increased TCDD exposure. In sprayed areas, a significantly increased OR in the high-TCDD group was observed. No significant associations, however, were found between having a girl and TEQ-PCDD/F levels. These results suggest that maternal TCDD exposure may alter the sex ratio at birth among Vietnamese residents of areas with high dioxin contamination.

## 1. Introduction

During the Vietnam War, over 71 million liters of herbicides such as Agent Orange, which contained large quantities of 2,3,7,8-tetrachlorodibenzo-p-dioxin (TCDD) [1,2], were sprayed across southern Vietnam below the former demilitarized zone at the 17th parallel.

In 2007 and 2010, more than 40 years after herbicide spraying had ceased, Hatfield Consultants and the Vietnamese government reported extremely high levels of dioxins in soil and sediment samples collected from Da Nang and Bien Hoa airbases due to the environmental long half-life (e.g., 25–100 years in subsurface soil) [3]. The maximum TCDD in soil and sediment samples (0–10 cm depth) was 365,000 ppt and 27,700 ppt, respectively, in Da Nang [4] and 61,400 ppt and 2020 ppt, respectively, in Bien Hoa [5]. In addition, we reported high dioxin levels in breast milk samples collected from nursing mothers residing in areas near former United States (US) airbases in Da Nang and Bien Hoa, Vietnam, compared to nursing mothers living in unsprayed areas [6,7]. In Da Nang and Bien Hoa, we conducted birth cohort studies to examine the effects of dioxin on infant neurodevelopment. We recruited mother–infant pairs at birth in Da Nang during 2008–2009 and in Bien Hoa during 2012 and 2015.

In the Ha Dong district of Hanoi city, located in the upper 17th parallel, we recruited mother–infant pairs in 2014 as a reference cohort living in an unsprayed area of Vietnam. As maternal exposure markers, we collected breast milk samples from nursing mothers in these three birth cohorts at 1 month after delivery, and we measured 17 congener levels of polychlorinated dibenzo-p-dioxins/dibenzofurans (PCDD/Fs) in breast milk. Moreover, we collected umbilical cord blood samples from mother–infant pairs in Bien Hoa and reported significant correlations of dioxin levels, particularly TCDD levels, in breast milk and cord blood samples [8], indicating dioxins in breast milk as good perinatal exposure markers.

The sex ratio at birth is widely used as an index reflecting alteration in the living environment and health status during pregnancy in terms of multiple factors such as paternal age and solar radiation, as reported in China by Jiang and Zhang (2021) [9], in the US by Sanchez-Barricarte (2023) [10], and in Poland by Nieczuja-Dwojacka (2024) [11]. As a marker of exposure to endocrine-disrupting chemicals (EDCs), the sex ratio has been used in epidemiological studies in Seveso, Italy investigating excess female births among offspring whose parents were accidentally exposed to high concentrations of TCDD, as indicated in serum levels [12,13]. A change in the sex ratio associated with dioxin exposure has been investigated in occupational populations [14,15] and Vietnam War veterans [16] exposed to TCDD. The results suggest that paternal TCDD exposure may have decreased the sex ratio among residents of Seveso, Italy and industrial workers; however, no association has been found between Agent Orange exposure and the sex ratio in offspring of American veterans. In cohort studies of mothers exposed to polychlorinated biphenyls (PCBs) through the consumption of contaminated fish, the adjusted odds ratio (OR) for having a boy in the highest-exposed mothers was significantly decreased, suggesting a lower sex ratio among offspring associated with maternal exposure [17,18,19].

We aimed to use logistic regression analysis to investigate the effects of high levels of maternal TCDD exposure and the toxic equivalent quantity of PCDD/Fs (TEQ-PCDD/Fs) on the sex ratio in Vietnamese children from three birth cohorts: in Da Nang and Bien Hoa, which are areas with high levels of dioxin contamination, as well as in the Ha Dong district in Hanoi city, an area that was not sprayed with herbicides during the Vietnam War.

## 2. Materials and Methods

### 2.1. Study Areas and Participants

The study area comprised two areas near Da Nang and Bien Hoa airbases in Vietnam with high dioxin contamination. After excluding infants with premature birth and low birth weight, a total of 453 mother–infant pairs were enrolled in this study, including 219 pairs from the Da Nang birth cohort recruited in 2008 and 2009 [20] and 254 pairs from the Bien Hoa birth cohort recruited in 2012 and 2015 [21]. For the unsprayed area, we enrolled 103 mother–infant pairs, without premature or low birth weight infants, from the Hanoi birth cohort recruited in 2014 [21]. Ha Dong is a residential area located in northern Vietnam that has not been affected by industrial pollution. The characteristics of mother–infant pairs in each area (total = 576) with testing results of comparisons between 3 areas are shown in Table 1. Characteristic indices in Da Nang and Bien Hoa were compared with those in Hanoi. The indices in Bien Hoa were also compared with those in Da Nang.

### 2.2. Dioxin Measurements in Breast Milk

At 1 month after birth, medical staff from community health stations in each area visited participating mothers at home and collected approximately 20 mL of breast milk with the mothers’ assistance. Milk samples were temporarily stored in freezers at −4 °C in local health stations, and frozen samples on dry ice were transferred to Kanazawa Medical University in Japan and stored at −30 °C until analysis of 2,3,7,8-substituted PCDD/Fs levels in breast milk.

The milk fat was extracted from each sample and its content was determined gravimetrically. As internal standards, 2,3,7,8-substituted, 13C-labeled PCDD/Fs (DF-LCS-A40, Wellington Laboratories Inc., Guelph, ON, Canada) were added to each sample. For extraction, purification, and fractionation, dioxin measurement grade reagents; hexane, acetone, dichloromethane, and toluene and pesticide residue PCB measurement grade reagents; diethyl ether and petroleum ether were used. Sorbent materials including silica gels, anhydrous sodium sulfate, and active carbon-dispersed silica gel [22] were also implemented. Quantification of 17 PCDD and PCDF congeners was performed using a gas chromatograph (HP-6980; Hewlett-Packard, Palo Alto, CA, USA) equipped with a high-resolution mass spectrometer (HR-GC/MS; MStation-JMS700, JEOL, Tokyo, Japan). The limit of detection (LOD) was achieved at a signal-to-noise (S/N) ratio = 3. If measurement values of each congener were below the LOD, half of the LOD was used as a quantification value. Recovery rates were within 50% to 118%. Details regarding the dioxin analysis of milk samples have been reported in our previous studies [6,7,23]. Additionally, for quality assurance in dioxin measurement, a reference material (CRM67; Community Bureau of preference BCR, European Commission, Belgium) comprising freeze-dried milk powder diluted with distilled water was measured regularly [6].

The TEQ-PCDD/Fs were calculated by summing the values of each congener concentration after it had been multiplied by its toxicity equivalence factor (TEF), with the World Health Organization 2005-TEF as reference [24].

The levels of TCDD and TEQ-PCDD/Fs in breast milk were used as dioxin exposure markers, which were stratified according to four levels (low, mild, moderate, and high) with cutoff values of 2.5, 3.5, and 5.5 (pg/g lipid) for TCDD and 8.5, 11.5, and 17.5 (pg-TEQ/g lipid) for TEQ-PCDD/Fs, respectively. The relationships between TCDD levels and TEQ-PCDD/Fs levels in Hanoi (unsprayed area) and in Da Nang and Bien Hoa (exposed areas) are shown in Table 2.

### 2.3. Statistical Analysis

IBM SPSS version 22.0 software (IBM Corp., Armonk, NY, USA) was used for the statistical analyses.

To clarify the associations between the sex ratio in offspring and dioxin exposure, ORs for having a girl among mothers with high exposure levels to TCDD or TEQ-PCDD/Fs were determined using a binary logistic regression model, after adjusting for maternal age, parity (primipara or multipara), and higher education level (14 years or more). Similarly, for mother–infant pairs with information on the father’s age, ORs for having a girl were determined after adjusting for the father’s age in addition to maternal confounding factors. The results were considered when *p* < 0.05 for all tests.

### 2.4. Supplementary Analysis: Paternal Exposure

In the current study, the effects of TCDD on the infant sex ratio associated with paternal exposure could not be investigated owing to a lack of exposure assessment data for all fathers in the birth cohorts. However, significant effects of paternal dioxin exposure on the sex ratio among offspring have been reported in previous studies [12,13,14,15]. We measured and reported blood dioxin levels and their relevant factors, such as a job related to an airbase, for only 40 fathers in Bien Hoa whose children were recruited in 2015 [25]. We linked data of dioxin levels in maternal breast milk with paternal dioxin levels in blood (N = 37) in the Bien Hoa birth cohort (2015) and analyzed the correlations between them to determine whether paternal exposure could be a confounding factor of maternal exposure. At this time, a non-parametric method (Spearman’s ρ) was used because of high skewness of dioxin parameters even after lognormal transforming (Supplementary Tables S1 and S2).

## 3. Results

As shown in Table 1, some characteristics of participants in the current study were different among the three study areas. Particularly, education levels and family income are lowest in Da Nang, followed by Bien Hoa. In Hanoi, women commonly have higher education levels (college and higher) compared with women in Da Nang and Bien Hoa.

The distributions of TCDD and TEQ-PCDD/Fs levels in breast milk in the three areas are shown in Table 2. All samples in Hanoi (unsprayed area) were included in the groups with low exposure to TCDD and TEQ-PCDD/Fs. More than 75% of samples in Da Nang and 63% of samples in Bien Hoa were included in the low-TCDD groups. Only 4% of samples in Da Nang but 13% of samples in Bien Hoa exhibited high levels of TCDD (≥5.5 pg/g lipid).

In contrast to TCDD, for TEQ-PCDD/Fs, 50% of samples in Bien Hoa but only 20% of samples in Da Nang were included in the low-exposure groups. Less than 10% of samples in Bien Hoa and more than 25% of samples in Da Nang had high levels of TEQ-PCDD/Fs (≥17.5 pg-TEQ/g lipid).

Table 3 shows the sex ratio indices at birth in each area and in exposed areas (Da Nang and Bien Hoa), including the ratio of male to female infants (M/F ratio) and the male proportion, according to exposure levels of TCDD and TEQ-PCDD/Fs. The M/F ratio and male proportion at birth in the low-TCDD groups were 1.55 and 0.608 in Da Nang and 1.15 and 0.534 in Bien Hoa, respectively, suggesting no decrease in the sex ratio in dioxin-exposed areas. This result was in comparison with the M/F ratio and male proportion of 1.02 and 0.505 in unexposed population in Hanoi and 1.06 and 0.515 in the general population [26], respectively. However, in the moderate- and high-TCDD groups in Da Nang and the mild-, moderate-, and high-TCDD groups in Bien Hoa, the sex ratio decreased with increased TCDD levels. Specifically, in the high-TCDD group, the M/F ratio and male proportion at birth were 0.29 and 0.222 in Da Nang and 0.65 and 0.394 in Bien Hoa, respectively, which were much lower than these values in the general population. When we compared sex ratio indices in the low-TCDD groups of all areas including Hanoi (1.25 and 0.556; not shown in Table 3), the high-TCDD group in all exposed areas showed much lower values, with 0.56 for the M/F ratio and 0.357 for the male proportion. Taken together, these results suggest a lower sex ratio at birth (excess female births) associated with TCDD exposure.

For TEQ-PCDD/Fs, the sex ratio indices (M/F ratio and male proportion) in high-exposure groups were 0.93 and 0.483 for Da Nang, 0.50 and 0.333 for Bien Hoa, and 0.80 and 0.443 for all exposed areas, respectively, which were lower than those in the low-exposure groups in each area. In both Da Nang and Bien Hoa, however, the mild- and moderate-exposure groups showed higher values for the sex ratio indices compared with the low-exposure group in each area, suggesting no clear association between decreased sex ratios and increased TEQ-PCDD/Fs levels (Table 3).

To clarify the associations of alterations in the sex ratio (increased number of girls) at birth and dioxin exposure levels after adjusting for maternal confounding factors, we analyzed the ORs for female birth associated with increased TCDD or TEQ-PCDD/Fs exposure levels in all areas, including the unsprayed area (Hanoi). We also analyzed the ORs for female birth only in the exposed (sprayed) areas, namely, Da Nang and Bien Hoa, because of the remarkable differences in maternal education levels between herbicide- sprayed and unsprayed areas. The results are shown in Table 4, adjusting for maternal factors including age, parity, mothers’ higher education level (14 years or more), and smoking among family members.

In all areas, the adjusted ORs (95% confidence interval [CI]) of female birth (reference: low-TCDD group) were 2.11 (1.05, 4.38) for the moderate-TCDD group and 2.77 (1.40, 5.49) for the high-TCDD group, which were significantly associated with increased TCDD exposure (*p* = 0.037 and *p* = 0.003, respectively). However, no significant associations were found between female birth and TEQ-PCDD/Fs levels.

Similarly, in sprayed areas, adjusted ORs were increased with increased TCDD levels, with an OR (95% CI) in the high-TCDD group of 2.64 (1.30, 5.35), indicating a significant association between female birth and high TCDD exposure. The adjusted OR in the high-TEQ-PCDD/Fs group was 1.72, but the significance between female birth and high TEQ-PCDD/Fs exposure was borderline (*p* = 0.077).

**Figure 1:**
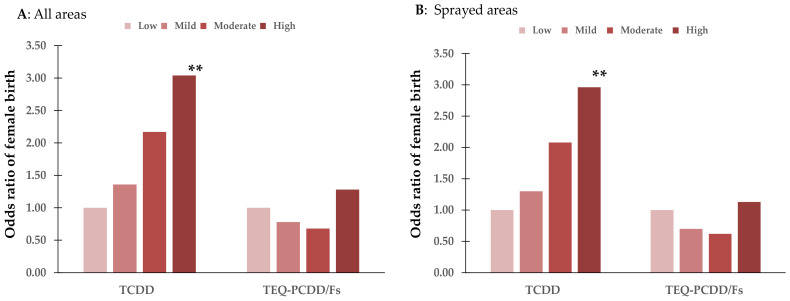
Associations between female birth and maternal dioxin exposure in all areas ((**A**): N = 507) and sprayed areas ((**B**): N = 408) after adjusting maternal confounding factors and fathers’ age. Maternal confounding factors: age, parity, family smoking, and higher education level (14 years and more), **: *p* < 0.01 compared with low-exposure group. P for trend: 0.012 for TCDD and 0.198 for TEQ-PCDD/Fs in all areas (**A**) and 0.019 for TCDD and 0.161 for TEQ-PCDD/Fs in sprayed areas (**B**).

After adding the confounding factor of the father’s age, similar analysis was performed for mothers with available information for the father’s age at birth (69 with missing data) to confirm the associations between female birth and dioxin exposure in all areas and sprayed areas (Figure 1).

In all areas (Figure 1A), the adjusted OR (95% CI) in the high-TCDD group was 3.04 (1.42, 6.53), which was significantly associated with high TCDD exposure (*p* = 0.004). The possibility of female birth was also associated with a high level of TCDD exposure in sprayed areas (*p* = 0.006), with an adjusted OR of 2.96 (95% CI 1.37, 6.37) (Figure 1B). However, no significant association was observed between female birth and TEQ-PCDD/F levels.

In addition, the correlation coefficients of 17 PCDD/F congener levels between maternal breast milk and paternal blood in 37 parents from the Bien Hoa cohort (2015) are shown in Supplementary Table S2. Concentrations of 1,2,3,6,7,8-hexachlorodibenzo-*p*-dioxin (1,2,3,6,7,8-HxCDD) in paternal blood were significantly correlated with levels of 1,2,3,6,7,8-HxCDD, TEQ-PCDDs, and TEQ-PCDD/Fs in maternal breast milk (ρ = 0.392, *p* = 0.017; ρ = 0.353, *p* = 0.032; and ρ = 0.333, *p* = 0.044, respectively), although no significant correlations were found between TCDD and TEQ-PCDD/Fs levels. These results suggest that 1,2,3,6,7,8-HxCDD may be the most closely correlated congener between father and mother pairs. Additionally, we reported that 1,2,3,6,7,8-HxCDD levels in breast milk were significantly correlated with food intake, particularly the intake of meat (*p* < 0.01), after adjusting confounding factors such as maternal age and residency in the Bien Hoa birth cohort recruited in 2012 [7]. Taken together, paternal exposure to 1,2,3,6,7,8-HxCDD might be a confounding factor in maternal exposure to 1,2,3,6,7,8-HxCDD because of the similar food intake in couples. However, no correlation with TCDD levels was detected for maternal and paternal exposure, suggesting that paternal TCDD exposure may not be a confounding factor in the association of maternal exposure to TCDD with increased female birth.

## 4. Discussion

In areas sprayed with herbicide in Vietnam, mothers with high TCDD exposure (≥5.5 pg/g lipid in breast milk) showed a three times higher probability of giving birth to a female infant, as compared with mothers with low TCDD levels (<2.5 pg/g lipid), after adjusting for confounding maternal factors and paternal age. These results suggest that maternal TCDD exposure influences the sex ratio at birth of offspring, resulting in an increased birth rate of female infants. However, this effect might be specific to TCDD and may not apply for other dioxin congeners because no significant effect of TEQ-PCDD/Fs on the sex ratio was identified.

### 4.1. Effects of TCDD Exposure on Sex Ratio

A lower sex ratio at birth associated with paternal TCDD exposure was reported in accidentally exposed residents of Seveso, Italy [12,13] and among herbicide producers in Russia [14] and New Zealand [15], whereas our results showed an association with maternal exposure to TCDD.

Excess female births (lower sex ratio at birth) were reported among offspring whose parents were accidentally exposed to high concentrations of TCDD in Seveso, Italy, in 1972 [12,13]. Mocarelli et al. (2000) [13] found a decreased offspring sex ratio to be associated with paternal exposure but not with maternal exposure, indicated by TCDD levels in sera collected 2 weeks after the plant explosion. Their results suggest a possibility that paternal exposure might confound associations between increased female births and maternal exposure found in the current study, because of significant correlations expected between maternal and paternal exposure levels due to their similar living style. However, in our preliminary correlation analysis of PCDD/F congener levels between maternal breast milk and paternal blood samples in 37 parents, no statistically significant correlation with TCDD levels was detected between them, suggesting that paternal TCDD exposure may not confound our findings of altered sex ratio at birth associated with maternal TCDD exposure indicated by breast milk levels.

In reports on industrial workers, authors detected no effect of maternal exposure on male birth. There were too few female exposed workers, however, in Russia [14] and in New Zealand [15] to obtain significant results after adjusting for confounding factors.

Additionally, in the current study, the effects of TCDD on the sex ratio at birth were analyzed while adjusting for factors relevant to increased female birth, which were found recently [8,9,10] and not used in the analysis of previous studies.

Taken together, maternal TCDD exposure, as well as paternal exposure, may influence the offspring sex ratio at birth.

For TCDD exposure originating from Agent Orange, two case–control studies targeting children of male US veterans [16] and male Agent Orange producers in the US [27] showed no effect of TCDD exposure on the sex ratio. The exposure assessment methods used in those studies might yield results that are inconsistent with those in studies among Seveso residents [12,13] and industrial workers [14,15]. For example, blood TCDD levels were measured at a later time after birth in all children, and TCDD exposure in each pregnancy was estimated using the history of service in Project Ranch Hand (herbicide spraying) in Vietnam [16], or serum TCDD was estimated using a pharmacokinetic model based on the working history in herbicide production [27].

### 4.2. Effects of Exposure to Other Endocrine Disrupting Chemicals (ERCs) on Sex Ratio

PCBs are major environmental contaminants with endocrine-disrupting properties, among which the most common and powerful is TCDD. Researchers have investigated populations that consume contaminated fish from a lake or ocean and the lower sex ratio among children in association with parental PCB exposure [17,18,19]. These reports describe altered male births associated with maternal exposure as well as with paternal exposure.

Among residents who consumed fish from the contaminated Great Lake in Michigan, USA, Karmaus et al. (2002) [17] reported a significant association between male births and serum PCB levels in fathers, with a more than two-times-higher OR in the high-exposure group (>8.1 μg/L), suggesting that increased male births are associated with paternal PCB exposure. In the same population, Weisskopf et al. (2003) [18] investigated male births associated with maternal exposure and reported significantly (82%) reduced ORs (lower male births) in the highest quantile exposure group (serum PCB > 3.0 μg/L) after adjusting for confounding factors including parental ages. Those authors also assessed the ORs associated with paternal PCB exposure levels, but no significantly increased ORs were found in the highest quantile group (serum PCB > 6.2 μg/L), revealing different results from those of Karmaus et al. (2002) [17]. Hertz-Piccitto et al. (2008) [19] investigated the association between male birth and maternal PCB concentrations in serum collected during pregnancy in a cohort of pregnant women in the San Fransico Bay area and reported significantly decreased male births (OR 0.45) in mothers with PCB > 90th percentile of total PCBs, using mothers who had PCBs < 10th percentile as a reference. Those authors suggested that maternal PCB exposure may prevent successful conception owing to the quality of male sperm or the greater vulnerability of male embryos, leading to miscarriage.

In these environmentally exposed populations, however, abundant PCB congeners were non-dioxin-like PCBs, which may disrupt sexual development through estrogenic mechanisms, whereas dioxin-like PCBs may have an influence via the interaction between aryl hydrocarbon receptor (AhR) and estrogen receptors (anti-estrogenic mechanisms). Therefore, an alteration in the sex ratio associated with PCB exposure might be caused by different mechanisms from TCDD exposure observed in the current study, because of the most typical AhR agonist for TCDD.

In Japanese women with Yu-sho disease (rice oil poisoning) who were heavily exposed to PCBs and PCDFs (dibenzofurans), no significant effects were found on the sex ratio of their offspring [28] or the frequency of spontaneous abortion during 36 years of observation, although induced abortion was significantly frequent in the first 10 years after exposure [29]. However, in a large-scale, population-based epidemiological study of infants born in Yu-sho-endemic areas, the male proportion at birth was significantly lower in the first 10 years after exposure [30]. These study findings suggest that the impact of PCBs and PCDFs on the sex ratio at birth may be detectable if information is collected during the early period after exposure and from a large number of affected individuals.

### 4.3. Possible Mechanism for Lower Sex Ratio Associated with Maternal TCDD Exposure

Few studies have investigated maternal factors altered by environmental dioxin exposure leading to an alteration in the sex ratio at birth, perhaps because of reports of lower male births significantly and specifically associated with paternal TCDD exposure in Seveso, Italy [13].

In industrialized countries, stress among women during pregnancy, including chemical stress, is believed to be one factor affecting the offspring sex ratio owing to the alteration of gonadotropin and testosterone levels [31]. This suggests that an altered hormonal environment among mothers associated with EDC exposure may influence the phenotypic determination of fetal sex. James (2020) [32] suggested that if, after conception, mothers are under stress in association with TCDD exposure (the most typical EDC), increased relative levels of testosterone may lead to increased fetal loss, particularly for a male fetus because of greater vulnerability than a female fetus. Jongbloet et al. (2004) [33] also indicated higher male fetal loss; but, through a mechanism different to that suggested by James [33], Y-bearing sperm tend to fertilize non-optimally ripe oocytes, forming zygotes with a higher attrition rate, suggesting the involvement of maternal TCDD exposure in a decreased male birth rate.

Among participants in the Seveso Women’s Health Study (SWHS), longer menstrual cycles were observed in women who were pre-menarche and were accidentally exposed to TCDD [34]; albeit, no significant alteration in ovarian function was found in association with serum TCDD levels [35]. However, clearer adverse effects on the ovaries, such as reduced ovulation, have been reported in animal studies [36]. Taken together, maternal exposure may influence the maturation of oocytes, which might lead to Y-bearing embryos being lost more frequently in these mothers than in mothers who are not exposed to dioxins, with increased female births in mothers exposed to TCDD.

In the SWHS, a longer time to pregnancy and higher infertility rate were found among women in the high-TCDD group [37]. These results are consistent with the adverse effects of TCDD in animal studies on fertility parameters, including time to pregnancy and maintenance of pregnancy [37], as well as on uterine functions including estrogen-mediated epithelial function [38] and successful implantation [39]. Yoshizawa et al. (2009) [40] also reported that TCDD induces inflammation in uterine tissue. These results suggest that TCDD might produce an inadequate uterine environment for Y-bearing embryos or a male fetus to survive during pregnancy.

### 4.4. Strength and Limitation

To the best of our knowledge, this is the first report to investigate the effects of maternal dioxin exposure originating from Agent Orange on the offspring sex ratio in the Vietnamese population based on quantified dioxin levels in breast milk using gas chromatography–mass spectrometry measurements [6,7,23]. In the current study, we analyzed ORs, after adjusting for maternal confounding factors including maternal educational levels, which were not adjusted in previous studies on the infant sex ratio at birth among US veterans [16] and industrial workers exposed to herbicides, including TCDD [14,15].

In a relatively recent, well-designed epidemiological study among industrial workers in New Zealand [15], the authors adjusted for several confounding factors including age, body mass index, and smoking status of the father or mother in an analysis of the effects of TCDD exposure on the infant sex ratio; however, no socioeconomic factors, such as educational levels, were adjusted for in that study. In the current study, we adjusted for the father’s age, which was significantly correlated with excess female births, independent of maternal age. We believe that adjusted analyses with sufficient confounding factors are important but rarely performed in local Vietnamese populations; this was a strength of our current study. However, the number of participants in the current analysis was limited, particularly in unsprayed areas, because of missing factors in the adjustment of confounders, including the preference for a son in Asian countries, which was not an issue for this study.

We used the same method to measure dioxin levels in breast milk just 1 month after birth in all areas, including unsprayed areas, as a maternal exposure marker during the current pregnancy. This exposure assessment yielded an accurate quantitative estimation of maternal exposure during pregnancy, which is another strength of our study.

However, this study has a major limitation in that we could not investigate the effects of paternal exposure on the infant sex ratio because of a small sample size and limited collection area (only Bien Hoa) for blood samples from fathers to conduct dioxin measurements. In the future, we aim to carry out exposure assessments of fathers according to dioxin categories and analyze the correlation between dioxins in maternal breast milk and paternal dioxin exposure markers, not only in Bien Hoa but also in Da Nang and Hanoi (unsprayed area), to clarify the contribution of paternal dioxin exposure to the sex of offspring in these Vietnamese birth cohorts.

## 5. Conclusions

We investigated the effects of maternal exposure on the sex ratio at birth in Vietnamese infants from three birth cohorts, including 576 mother–infant pairs in herbicide-sprayed and unsprayed areas, after adjusting for confounding factors including maternal education and father’s age. Maternal TCDD was significantly associated with increased female births, with ORs of 2.1–3.0 in high-TCDD groups (≥5.5 pg/g lipid), suggesting that maternal TCDD exposure may influence the sex ratio at birth.

## Figures and Tables

**Table 1:** Characteristics of participants in Hanoi (unsprayed area) and Da Nang and Bien Hoa (dioxin-exposed areas).

Areas		Hanoi		Da Nang		*p*	Bien Hoa		*p*
N of pairs		103		219			254		
Mothers									
Age (years)	mean, SD	27.1	3.6	27.9	6.0		28.8	5.1	***
Parity (% of primipara)	N, %	42	38.9	65	29.7		105	41.5	##
Education (years)	mean, SD	14.9	1.8	8.6	3.5	***	11.1	2.7	***, ###
(higher education: >14 years)	N, %	92	85.2	22	10.0	***	28	11.1	***
Alcohol drinking (yes, no)	N, %	8	7.5	40	18.3	*	6	2.4	*, ###
Family income (×1000 VND/month)	mean, SD	13.6	9.6	3.0	1.6	***	10.4	10.6	*, ###
Family smoking (yes, no)	N, %	44	42.7	181	82.6	***	160	63.2	###
Fathers									
Age (years)	mean, SD	31.1	4.6	31.5	6.1		32.1	5.9	

The number of fathers was a total of 507 including 99 for Hanoi, 187 for Da Nang, and 221 for Bien Hoa. N: number of subjects, SD: standard deviation, VND: Vietnamese dong, *p*: *p*-value. *: *p* < 0.05, ***: *p* < 0.001 compared with Hanoi, ##: *p* < 0.01, ###: *p* < 0.001 compared with Da Nang.

**Table 2:** TCDD and TEQ-PCDD/Fs levels in maternal breast milk of birth cohorts in herbicide-sprayed and unsprayed areas.

Dioxin Exposure	Unsprayed Areas		Sprayed Areas					
	Hanoi		Da Nang		Bien Hoa		All Exposed Areas	
	N = 103		N = 219		N = 254		N = 473	
	N	(%)	N	(%)	N	(%)	N	(%)
TCDD (pg/g lipid)								
Low (<2.5)	103	(100.0)	171	(78.1)	161	(63.4)	332	(70.2)
Mild (2.5–3.5)	0	(0.0)	27	(12.3)	37	(14.6)	64	(13.5)
Moderate (3.5–5.5)	0	(0.0)	12	(5.5)	23	(9.1)	35	(7.4)
High (≥5.5)	0	(0.0)	9	(4.1)	33	(13.0)	42	(8.9)
TEQ-PCDD/Fs (pg-TEQ/g lipid)								
Low (<8.5)	103	(100.0)	45	(20.5)	127	(50.0)	172	(36.4)
Mild (8.5–11.5)	0	(0.0)	39	(17.8)	61	(24.0)	100	(21.1)
Moderate (11.5–17.5)	0	(0.0)	77	(35.2)	45	(17.7)	122	(25.8)
High (≥17.5)	0	(0.0)	58	(26.5)	21	(8.3)	79	(16.7)

N: number of subjects.

**Table 3:** The ratio of male to female infants and male proportion at birth in the herbicide-sprayed and unsprayed areas.

	Unsprayed				Sprayed Areas											
	Hanoi				Da Nang				Bien Hoa				All Exposed Areas			
	N of Male	N of Female	Male/Female	Male Proport	N of Male	N of Female	Male/Female	Male Proport	N of Male	N of Female	Male/Female	Male Proport	N of male	N of Female	Male/Female	Male Proport
TCDD																
Low	52	51	1.02	0.505	104	67	1.55	0.608	86	75	1.15	0.534	190	142	1.34	0.572
Mild	0	0		-	14	13	1.08	0.519	18	19	0.95	0.486	32	32	1.00	0.500
Moderate	0	0		-	6	6	1.00	0.500	8	15	0.53	0.348	14	21	0.67	0.400
High	0	0		-	2	7	0.29	0.222	13	20	0.65	0.394	15	27	0.56	0.357
TEQ-PCDD/Fs																
Low	52	51	1.02	0.505	25	20	1.25	0.555	63	64	0.98	0.496	88	84	1.05	0.512
Mild	0	0		-	27	12	2.25	0.692	32	29	1.10	0.525	59	41	1.44	0.590
Moderate	0	0		-	46	31	1.48	0.597	23	22	1.05	0.511	69	53	1.30	0.566
High	0	0		-	28	30	0.93	0.483	7	14	0.50	0.333	35	44	0.80	0.443

N: number of subjects, Male/Female: the ratio of male to female infants at birth, Male proport: Male proportion at birth.

**Table 4:** Associations between female birth and maternal dioxin exposure after adjusting maternal confounding factors.

		N	Female Birth	Odds Ratio	95% CI		*p*-Value
					Upper	Lower	
All areas (N = 576)							
TCDD	Low	435	193	1.00	(p for trend = 0.007)		
	Mild	64	32	1.45	0.84	2.49	0.182
	Moderate	35	21	2.11	1.05	4.38	0.037
	High	42	27	2.77	1.40	5.49	0.003
TEQ-PCDD/Fs	Low	275	135	1.00	(p for trend = 0.228)		
	Mild	100	41	0.82	0.50	1.33	0.417
	Moderate	122	53	0.89	0.57	1.41	0.616
	High	79	44	1.46	0.87	2.54	0.146
Sprayed areas (N = 473)							
TCDD	Low	332	142	1.00	(p for trend = 0.036)		
	Mild	64	32	1.38	0.76	2.50	0.295
	Moderate	35	21	1.71	0.81	3.59	0.157
	High	42	27	2.64	1.30	5.35	0.007
TEQ-PCDD/Fs	Low	172	84	1.00	(p for trend = 0.266)		
	Mild	100	41	0.92	0.54	1.60	0.777
	Moderate	122	53	1.14	0.67	1.94	0.630
	High	79	44	1.72	0.94	3.13	0.077

N: number, CI: confidence interval. Confounding factors: maternal age, parity, family smoking, and higher education (14 years and more).

## Data Availability

The data presented in this study are available upon request to the corresponding author.

## Supplementary Materials

The following supporting information can be downloaded at: https://www.mdpi.com/article/10.3390/toxics12040276/s1, Table S1: Skewness and Kurtosis of dioxin markers of paternal and maternal exposure (37 pairs); Table S2: Correlation coefficients (Spearman’s rho) between dioxin congeners and TEQs between maternal and paternal exposures (37 pairs).
