# Supplementary material for: Altered Sex Ratio at Birth with Maternal Exposure to Dioxins in Vietnamese Infants
Source: Toxics. 2024 Apr 9;12(4):276. doi: 10.3390/toxics12040276 (PMC11053781; doi:10.3390/toxics12040276)
Supplement: Supplementary file 1 [file toxics-12-00276-s001.zip › toxics-2873662-supplementary.pdf]

Supple. Table 1 Skewness and Kurtosis of dioxin markers of paternal and maternal exposure (37 pairs)

| | Paternal exposure (lognormal transformed data) | | | | | | Maternal exposure (lognormal transformed data) | | | | | |
|---|---|---|---|---|---|---|---|---|---|---|---|---|
| | Min. | Max. | Mean | SD | Skewness | Kurtosis | Min. | Max. | Mean | SD | Skewness | Kurtosis |
| *PCDD congeners (pg/g lipid)* | | | | | | | | | | | | |
| 2,3,7,8-TCDD | 0.18 | 2.57 | 0.89 | 0.43 | 1.70 | 5.49 | -0.25 | 1.65 | 0.32 | 0.37 | 1.31 | 3.34 |
| 1,2,3,7,8-PeCDD | 0.64 | 1.35 | 1.02 | 0.17 | -0.18 | -0.58 | -0.06 | 0.69 | 0.40 | 0.16 | -0.64 | 0.81 |
| 1,2,3,4,7,8-HxCDD | 0.43 | 1.07 | 0.77 | 0.16 | -0.29 | -0.41 | -0.29 | 0.49 | 0.11 | 0.20 | 0.06 | -0.24 |
| 1,2,3,6,7,8-HxCDD | 0.74 | 1.57 | 1.12 | 0.18 | -0.02 | 0.37 | 0.24 | 1.08 | 0.54 | 0.20 | 0.64 | 0.07 |
| 1,2,3,7,8,9-HxCDD | 0.43 | 1.50 | 0.83 | 0.22 | 0.60 | 1.47 | -0.20 | 0.64 | 0.15 | 0.21 | 0.22 | -0.35 |
| 1,2,3,4,6,7,8-HeptaCDD | 0.97 | 2.47 | 1.52 | 0.28 | 0.89 | 2.93 | 0.37 | 1.35 | 0.86 | 0.22 | -0.09 | -0.37 |
| OCDD | 2.53 | 3.42 | 2.98 | 0.23 | 0.15 | -0.75 | 1.04 | 2.26 | 1.78 | 0.26 | -0.38 | 0.46 |
| *PCDF congeners (pg/g lipid)* | | | | | | | | | | | | |
| 2,3,7,8-TCDF | 0.17 | 1.00 | 0.63 | 0.20 | -0.33 | -0.31 | -0.70 | 0.22 | -0.20 | 0.24 | 0.03 | -0.64 |
| 1,2,3,7,8-PeCDF | 0.12 | 1.01 | 0.67 | 0.22 | -0.76 | 0.11 | -0.71 | 0.31 | -0.15 | 0.24 | -0.14 | -0.45 |
| 2,3,4,7,8-PeCDF | 0.82 | 1.34 | 1.13 | 0.13 | -0.13 | -0.44 | 0.12 | 0.96 | 0.46 | 0.16 | 1.05 | 2.29 |
| 1,2,3,4,7,8-HxCDF | 0.86 | 1.48 | 1.18 | 0.16 | -0.18 | -0.23 | 0.21 | 1.30 | 0.69 | 0.21 | 0.73 | 2.03 |
| 1,2,3,6,7,8-HxCDF | 0.46 | 1.28 | 1.02 | 0.17 | -1.03 | 1.67 | 0.07 | 1.04 | 0.42 | 0.19 | 0.95 | 2.37 |
| 1,2,3,7,8,9-HxCDF | -0.03 | 1.83 | 0.56 | 0.31 | 1.73 | 7.00 | -0.87 | 0.24 | -0.28 | 0.27 | -0.11 | -0.43 |
| 2,3,4,6,7,8-HxCDF | 0.10 | 0.99 | 0.59 | 0.20 | -0.21 | 0.37 | -0.56 | 0.34 | -0.17 | 0.21 | 0.14 | -0.04 |
| 1,2,3,4,6,7,8-HpCDF | 0.72 | 1.70 | 1.14 | 0.19 | 0.17 | 0.86 | -0.08 | 0.95 | 0.36 | 0.21 | 0.49 | 1.21 |
| 1,2,3,4,7,8,9-HpCDF | 0.04 | 1.18 | 0.68 | 0.29 | -0.21 | 0.13 | -0.69 | 0.26 | -0.26 | 0.27 | 0.26 | -0.75 |
| OCDF | 0.35 | 1.33 | 1.01 | 0.22 | -1.15 | 1.28 | -0.46 | 0.96 | 0.21 | 0.33 | -0.21 | -0.20 |
| *TEQs (pg-TEQ/g lipid)* | | | | | | | | | | | | |
| TEQ-PCDDs | 0.96 | 2.60 | 1.38 | 0.28 | 2.44 | 9.85 | 0.34 | 1.70 | 0.76 | 0.24 | 1.72 | 5.92 |
| TEQ-PCDFs | 0.70 | 1.15 | 0.93 | 0.11 | -0.18 | -0.52 | 0.01 | 0.76 | 0.28 | 0.16 | 1.36 | 3.12 |
| TEQ-PCDD/Fs | 1.16 | 2.60 | 1.52 | 0.24 | 2.70 | 11.55 | 0.52 | 1.75 | 0.89 | 0.21 | 2.06 | 7.30 |

Min.: minimum, Max.: maximum, SD: standard deviation

Suppl. Table 2 Correlation coefficients (Spearman's rho) between dioxin congeners and TEQs between maternal and paternal exposures (37 pairs).

| **Maternal exposure** | **Paternal exposure** | | | | | | | | | |
|---|---|---|---|---|---|---|---|---|---|---|
| *PCDD congeners* | 2,3,7,8-TCDD | 1,2,3,7,8-PeCDD | 1,2,3,4,7,8-HxCDD | 1,2,3,6,7,8-HxCDD | 1,2,3,7,8,9-HxCDD | 1,2,3,4,6,7,8-HpCDD | OCDD | | | |
| 2,3,7,8-TCDD | **0.157** | -0.053 | -0.029 | 0.246 | 0.050 | 0.050 | 0.107 | | | |
| 1,2,3,7,8-PeCDD | 0.005 | **-0.048** | -0.087 | 0.219 | 0.048 | 0.108 | 0.041 | | | |
| 1,2,3,4,7,8-HxCDD | 0.070 | 0.088 | **-0.043** | 0.280 | 0.065 | 0.080 | -0.133 | | | |
| 1,2,3,6,7,8-HxCDD | 0.064 | 0.189 | -0.087 | **0.392** * | -0.006 | 0.174 | 0.054 | | | |
| 1,2,3,7,8,9-HxCDD | -0.174 | 0.030 | -0.086 | 0.176 | **-0.034** | 0.081 | -0.082 | | | |
| 1,2,3,4,6,7,8-HpCDD | 0.019 | 0.184 | -0.075 | 0.322 | -0.005 | **0.293** | 0.107 | | | |
| OCDD | -0.020 | -0.053 | -0.290 | 0.031 | -0.23 | -0.128 | **-0.192** | | | |
| *PCDF congeners* | 2,3,7,8-TCDF | 1,2,3,7,8-PeCDF | 2,3,4,7,8-PeCDF | 1,2,3,4,7,8-HxCDF | 1,2,3,6,7,8-HxCDF | 1,2,3,7,8,9-HxCDF | 2,3,4,6,7,8-HxCDF | 1,2,3,4,6,7,8-HpCDF | 1,2,3,4,7,8,9-HpCDF | OCDF |
| 2,3,7,8-TCDF | **-0.224** | 0.021 | 0.119 | 0.187 | 0.295 | -0.100 | 0.107 | 0.237 | 0.055 | 0.021 |
| 1,2,3,7,8-PeCDF | -0.109 | **0.207** | 0.267 | 0.124 | 0.298 | -0.039 | 0.143 | 0.162 | -0.055 | 0.148 |
| 2,3,4,7,8-PeCDF | 0.262 | 0.043 | **0.093** | 0.205 | 0.043 | 0.197 | 0.072 | -0.042 | 0.468 ** | 0.171 |
| 1,2,3,4,7,8-HxCDF | -0.041 | -0.259 | -0.213 | **-0.092** | -0.248 | -0.094 | -0.249 | -0.046 | 0.18 | -0.030 |
| 1,2,3,6,7,8-HxCDF | -0.019 | -0.149 | -0.059 | 0.102 | **-0.110** | -0.031 | -0.151 | -0.025 | 0.257 | 0.076 |
| 1,2,3,7,8,9-HxCDF | -0.08 | 0.293 | 0.181 | 0.042 | 0.076 | **-0.005** | 0.225 | 0.296 | -0.035 | 0.230 |
| 2,3,4,6,7,8-HxCDF | -0.156 | -0.079 | 0.197 | 0.213 | 0.150 | -0.084 | **-0.028** | 0.035 | -0.063 | -0.011 |
| 1,2,3,4,6,7,8-HpCDF | -0.007 | -0.299 | -0.229 | -0.035 | -0.215 | -0.022 | -0.27 | **-0.014** | 0.072 | 0.000 |
| 1,2,3,4,7,8,9-HpCDF | -0.070 | 0.252 | 0.013 | -0.132 | -0.049 | 0.019 | 0.109 | 0.221 | **-0.023** | 0.190 |
| OCDF | 0.018 | 0.226 | 0.041 | 0.01 | 0.024 | 0.061 | 0.091 | 0.332 * | -0.169 | **0.166** |
| *TEQs* | TEQ-PCDDs | TEQ-PCDFs | TEQ-PCDD/Fs | | | | | | | |
| TEQ-PCDDs | **0.131** | -0.128 | 0.09 | | | | | | | |
| TEQ-PCDFs | 0.108 | **0.035** | 0.131 | | | | | | | |
| TEQ-PCDD/Fs | 0.147 | -0.052 | **0.123** | | | | | | | |
